# Development of an integrated fingerstick blood self-collection device for radiation countermeasures

**DOI:** 10.1371/journal.pone.0222951

**Published:** 2019-10-16

**Authors:** Jian Gu, Alan Norquist, Carla Brooks, Mikhail Repin, Sanjay Mukherjee, Jerome Lacombe, Jianing Yang, David J. Brenner, Sally Amundson, Frederic Zenhausern

**Affiliations:** 1 Center for Applied NanoBioscience and Medicine, The University of Arizona, College of Medicine, Phoenix, AZ, United States of America; 2 Center for Radiological Research, Columbia University, Vagelos College of Physicians and Surgeons, New York, NY, United States of America; University of Houston, UNITED STATES

## Abstract

We report the development of system for packaging critical components of the traditional collection kit to make an integrated fingerstick blood collector for self-collecting blood samples of 100 μl or more for radiation countermeasures. A miniaturized vacuum tube system (VacuStor system) has been developed to facilitate liquid reagent storage, simple operation and reduced sample contamination. Vacuum shelf life of the VacuStor tube has been analyzed by the ideal gas law and gas permeation theory, and multiple ways to extend vacuum shelf life beyond one year have been demonstrated, including low temperature storage, Parylene barrier coating and container vacuum bag sealing. Self-collection was also demonstrated by healthy donors without any previous fingerstick collection experience. The collected blood samples showed similar behavior in terms of gene expression and cytogenetic biodosimetry assays comparing to the traditionally collected samples. The integrated collector may alleviate the sample collection bottleneck for radiation countermeasures following a large-scale nuclear event, and may be useful in other applications with its self-collection and liquid reagent sample preprocessing capabilities.

## Introduction

In the 21^st^ century, nuclear terrorism is still a real and urgent threat [1]. In a large-scale nuclear event in a metropolitan area, it is estimated over a million people would seek information on their exposure levels [2]. Biodosimetry is a critical component in patient triage and management in radiation countermeasures [3]. However, rapid screening of hundreds of thousands of patients in a matter of a few days poses a great challenge to the existing biodosimetry infrastructure. To address this issue, efforts have been made to automate assay protocols for high-throughput screening [4] and specific protocols have also been accelerated to reduce the sample-to-answer time [5], but little attention has been paid to the process of sample collection so far.

Blood is a sample type used for many biodosimetry assays [3] due to its rich content and minimal invasiveness for sample collection. However, traditional blood collection using venipuncture can present a great bottleneck in collecting hundreds of thousands of samples quickly in radiation response due to the requirement for trained medical personnel who may not be available in the chaotic aftermath of a nuclear event. To meet the required surge in capacity, a self-collection device is highly desired.

Fingerstick blood collection requires less training and may be self-administered, but there are still multiple challenges to use existing fingerstick protocols for self-collection in a radiation emergency. In this paper, we will describe the rationale, design and prototyping of an integrated blood collector. We will also show the development of a critical subsystem of the collector, a miniature vacuum tube system, to be referred to as the “VacuStor” system. Ideal gas law was demonstrated for threshold vacuum pressure prediction, and different methods to extend the vacuum shelf life of VacuStor tubes longer than a year were also demonstrated, including low temperature storage, Parylene barrier coating and container vacuum bag sealing. With the developed collector, we successfully tested self-collection of finger pick blood, and performances of gene expression and cytogenetic biodosimetry assays using self-collected samples by the collectors are similar to those using traditionally collected samples.

## Integrated blood collector rationale and design

We are developing a collector for simultaneous blood self-collection for multiple biodosimetry assays, including two complementary assays, gene expression assay and cytokinesis-block micronucleus assay (CBMN) [3]. The gene expression assay has a much shorter sample-to-answer time (several hours), but is only valid for the first 1–7 days after the nuclear incident. The cytogenetic CBMN assay can be valid up to 3–6 months after the nuclear event, but it takes over 3 days for the first results to be available. To collect blood samples for the two assays, a predetermined volume of blood (~ 50–100 μl) needs to be collected for each assay. In addition, the collected blood samples need to be pre-processed after collection for each assay. An RNA stabilization solution is used to lyse the blood and stabilize RNA right after collection for the gene expression assay, and a cell culture medium is used to start blood cell culture right after collection and during transportation for the CBMN assay (a novel approach under investigation to dramatically reduce the time required for CBMN assay to generate the first dose result).

The components in a traditional fingerstick collection kit for the two assays would be 1) a lancet to prick finger, 2) two capillaries with desired anticoagulants to collect, measure minute volumes of blood, and transfer blood to designated sample tubes, 3) two sample tubes compatible with high-throughput liquid handling with desired reagents in each tube, 4) an optional hand warmer to reach large blood volume if needed, 5) an alcohol wipe for finger disinfection, 6) another alcohol wipe (or a sterile gauze pad) to clean the finger after collection, and 7) a BAND-AID to help wound healing and prevent infection.

We foresee three main challenges of the traditional kit for self-collection of blood for the biodosimetry assays. First, there are multiple small items in the kit (e.g. the capillaries and tube caps) that can be easily scattered, contaminated and/or lost during the collection process in the chaotic situation after a nuclear event. Second, a total collected blood volume of 100 to 200 μl will require a finger milking action by the non-pricked hand of the patient that will prevent him/her from handling the capillaries simultaneously for blood self-collection. Finally, during the collection process, the sample tube caps need to be removed for blood transfer, which increases the chance of liquid reagent spill and sample contamination.

**Fig 1:**
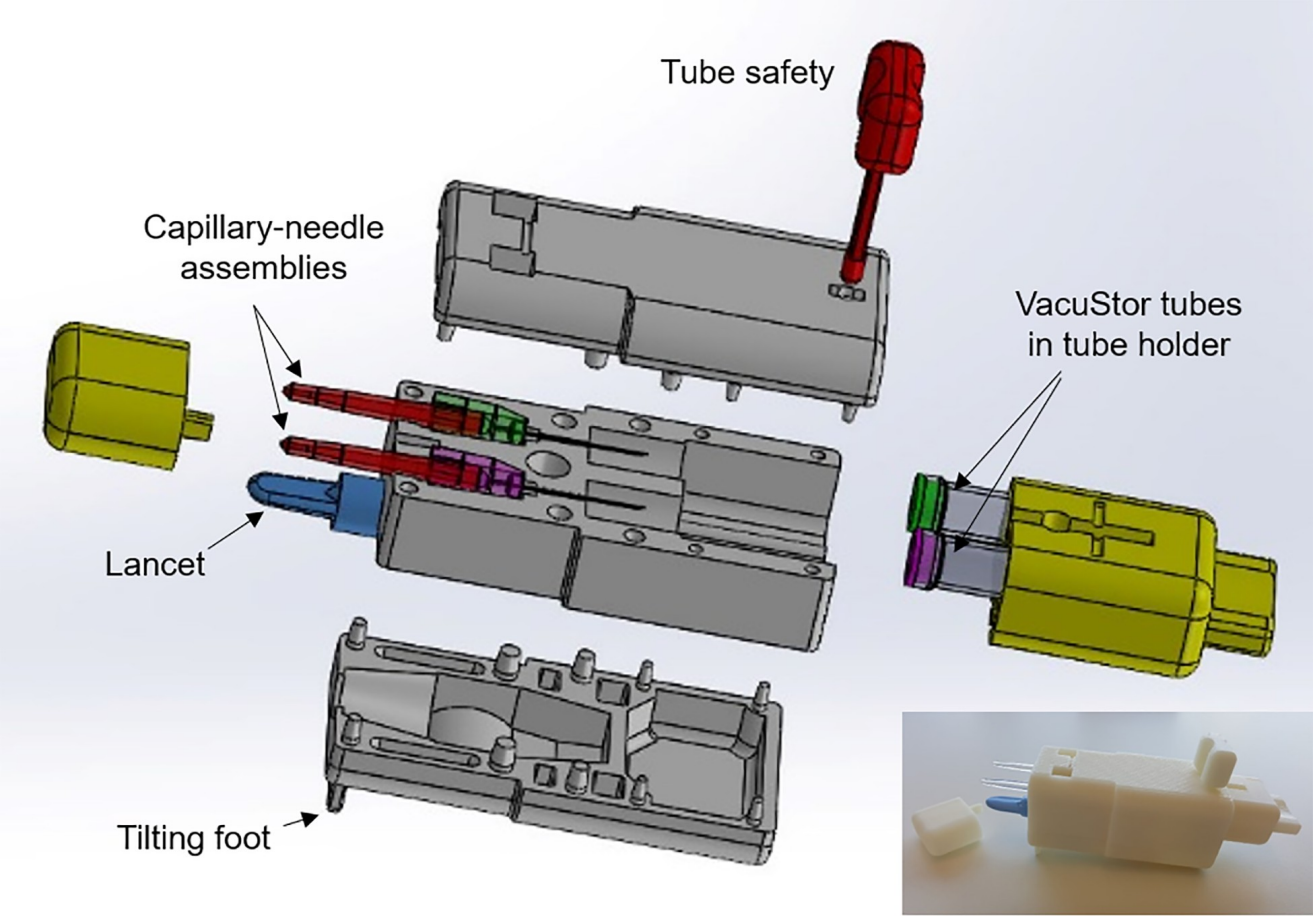
Design and prototyping of the integrated blood collector. Exploded view of the integrated blood collector, where one lancet, two capillary-needle assemblies and two VacuStor tubes are integrated by 3D printed parts; the insert shows an integrated blood collector prototype.

To address these challenges, we conceived the concept of an integrated blood collector, as shown in Fig 1. The integrated blood collector uses three enveloping layers to hold key components of the kit together (currently kit items 1–3 –more can be integrated in the future) to prevent loss/scattering. Furthermore, a single foot at the bottom of the device is used to tilt the tips of the capillaries up when the device sits on a flat table surface to aid the blood flowing into the capillaries without the need to handle the device. Finally, a VacuStor system was developed to reduce the handling steps and enable sealed liquid-reagent storage during collection to minimize possibilities for spill and contamination. The VacuStor system consists of a capillary-needle assembly and a VacuStor tube. The VacuStor tube is a pre-vacuumed storage tube containing the required liquid reagent. After a blood sample is collected into the capillary, the VacuStor tube cap is pierced by the needle, and the vacuum will draw the blood into the tube. The reason we chose the vacuum tube design for our integrated blood collector is that vacuum tubes have long been used for blood collection and have demonstrated significant advantages in collection safety, speed and sample integrity [6]. They also have liquid storage capability. Comparing with other integrated microfluidic reagent storage approaches reported before, such as glass ampoules and pouches/blisters [7–9], we believe the vacuum tube approach is more convenient and more compatible with the high-throughput liquid handling systems used in the central laboratory for the biodosimetry assays.

## Materials and methods

### Ethics statement

All blood collections were approved by the institutional review boards at The University of Arizona (IRB# 1708743060) and Columbia University (IRB# AAAF2671), and performed after written informed consents were given.

### Integrated blood collector prototyping

Geometries of a fingerstick lancet (BD Microtainer® Contact-Activated Lancet, blue), two capillary-needle assemblies and two glass VacuStor tubes were measured for the fabrication of the collector parts. The fabrication of the capillary-needle assembly and VacuStor tube is described in the next section. The enveloping layers, tube holder with safety, and capillary cap were printed by a uPrint SE Plus 3D printer (Stratasys, MN). Press fitting was done by a Carver Press (Wabash, IN) at room temperature. The capillary tilting angle was tested by taping the capillary on a stand under different tilting angle and testing the fluid intake speed and air gap formation using dyed phosphate buffered saline (PBS).

### Fabrication of capillary-needle assembly and VacuStor tube

The blood collecting capillaries were either 50 μl or 100 μl, purchased and used as is (Microvette 100 collection capillary, Sarstedt Inc) or cut from existing commercial products (Minivette 50μl or 100μl capillary, Sarstedt Inc). Anticoagulant coating of the capillary complies with the target assay protocol, i.e. potassium-EDTA for gene expression assay, and Lithium Heparin for CBMN assay. We purchased 27G 1.5” long hypodermic needles from EXELINT International Medical Products. The cannula of the needle was removed from the hub and ground to the appropriate length. Both the capillary and needle cannula were UV glued to an adaptor to complete the capillary-needle assembly. The adaptor was 3D printed using a Form2 SLA printer from Formlabs. After final assembly the needle cannula was cleaned with a sterile cotton swab and isopropyl alcohol. A quality control measure was introduced at a later stage of the project to identify clogged assemblies by passing compressed air through the assemblies.

VacuStor tubes were made using Matrix 2D barcoded 1 ml open top glass storage tubes from ThermoFisher Scientific Inc. (Item# 3850) that are in 96-rack format and compatible with high-throughput liquid handling. The caps were SepraSeal caps from the same company (Item# 4464). For individual VacuStor tube fabrication, a vacuum fixture with a gauge (SMC GZ43-K-01, which measures vacuum relative the environmental pressure) and a feedthrough was built to seal the VacuStor tubes with the desired vacuum. For high-throughput sealing of 96 VacuStor tubes simultaneously, a custom fixture and a chamber vacuum sealer (ARY VacMaster VP210) were used (see the 1^st^ section of S1 File for details).

### VacuStor system characterization

#### Threshold vacuum testing

To experimentally test the threshold vacuum for the case of 300 μl of liquid reagent and 100 μl of sample, VacuStor tubes containing 300 μl of PBS at different levels of vacuum were produced as described in 3.2. The environmental pressure P_env_ was measured by a digital barometer (VWR, Cat# 10510–922). The capped VacuStor tube volume (V_t_) was measured by pipetting 1 ml of PBS and measuring the remaining space dimensions of the tube.

With the VacuStor tubes ready, capillary-needle-assembles with cylindrical 100 μl capillaries (Microvette 100, Sarstedt Inc) were used to collect 100 μl of dyed PBS, then pierce the rubber cap of the VacuStor tubes at a horizontal position to transfer the samples to the tubes. The percentage of the sample transfer was measured by the ratio of emptied capillary length to the total capillary length (35 mm). Three experiments were conducted for each vacuum point.

#### Vacuum shelf life

To characterize the vacuum shelf life of the VacuStor tubes, a hole was drilled in the bottom of the glass tube by sand blasting, then the tube bottom was glued to the SMC vacuum gauge using a low outgassing epoxy glue (Torr Seal^®^ from Kurt J. Lesker Company). A similar fixture for sealing individual VacuStor tubes was used to cap the tube-gauge assembly under vacuum. Then the vacuum of the tube-gauge assembly was read out over time from the gauge. For low temperature experiments, once the tube-gauge assembly was formed and put into the desired environment, the assembly was allowed to stabilize before data collection. For Parylene coated caps, 5, 9 or 15.2 μm of Parylene C coating was applied to the SepraSeal caps by a SCS Labcoater^®^ 2 machine (Specialty Coating Systems, Indianapolis, IN) to form the VacuStor-gauge assembly and monitored at room temperature. The leaking time constants of the tube-gauge assembly (t_n_’ and t_o_’) were fitted by the least squares method using GRG Nonlinear Solver from Excel. Because the vacuum gauge has an internal volume (V_gauge_) that is connected with the tube, the real tube time constants t_n_ and t_o_ were converted from t_n_’ and t_o_’ using equation *t*_*i*_ = *t*_*i*_′*V_*t*_/(*V*_*t*_+*V*_*gauge*_) where i = n, o. V_gauge_ was measured to be 1450 μl by gluing a syringe to the gauge, then pulling the syringe to a certain volume and calculating V_gauge_ using the ideal gas law from the syringe volume and the vacuum readings from the gauge. After getting t_n_ and t_o_, shelf life can be found numerically using Eq (3) and the initial and threshold vacuums.

For vacuum bag sealing of the VacuStor tubes, 96 VacuStor tubes were first fabricated using Matrix 2D barcoded 1.4 ml polypropylene storage tubes (ThermoFisher Scientific Inc., Item# 3792) and SepraSeal caps. The rack container with the tubes were then sealed using the VacMaster VP210 vacuum sealer and a commercial 3-mil vacuum bag (ARY). A data logger (MRS145, MSR Electronics GmbH, Switzerland) was also sealed with the tube rack to measure the vacuum decay of the intermediate container space.

### Self-collection testing

To test self-collection of the blood collector, ten devices were fabricated as described. The capillaries used for the capillary-needle-assembles were cut from the 50 μl Minivette capillaries (Sarstedt Inc). The VacuStor tubes contained 300 μl PBS in place of required assay liquid reagents. The whole collection kit included 1) an integrated blood collector, 2) a hand warmer (McKesson Instant Hot Compress, cat# 16–9706), 3) an alcohol wipe (First Aid Only H305-200), 4) a Curad Small gauze pad, and 5) a BAND-AID (Johnson&Johnson). An educational video about conducting self-collection using the collector was made to illustrate the self-collection procedure (see S2 File).

### Gene expression and CBMN biodosimetry assays

#### Gene expression assay

300 μl of PAXgene Blood RNA solution (Qiagen) was used in both microcentrifuge tubes and VacuStor tubes for RNA stabilization. Total RNA was isolated from the collected blood using the PAXgene Blood RNA Kit (Qiagen) following manufacturer’s instruction. RNA was quantified using a Nanodrop ND1000 spectrophotometer (ThermoFisher Scientific) and RNA quality was checked by the 2100 Bioanalyzer (Agilent). Sodium-citrate tubes [Becton Dickinson (BD) Catalog-363083] were used for venous blood collection. A gamma source [Gammacell 40 ^137^Cesium irradiator (Atomic Energy of Canada, Ltd., Canada)] at a dose rate of 0.7 Gy/min was used for irradiation. The irradiated samples and the control were incubated at room temperature for 2h after irradiation before downstream processes. For gene expression comparison, RNA (200 ng) was used for cDNA synthesis using the High-Capacity cDNA Archive Kit (Life Technologies). The cDNA was used for real-time quantitative reverse transcript polymerase chain reaction (qRT-PCR) based gene expression analysis using the TaqMan chemistry. Gene expression analysis was carried out for 5 known radiation responsive genes. The gene expression assays (primer/probe sets) were purchased from ThermoFisher Scientific for the following genes: *CDKN1A* (Hs99999142_m1); *BAX* (Hs00180269_m1); *DDB2* (Hs03044953_m1); *FDXR* (Hs00244586_m1); *MDM2* (Hs01066938_m1), *ACTB* (Hs99999903_m1). The ΔΔCT method was used to calculate expression relative to controls, normalized against *ACTB* gene expression.

#### CBMN assay

PB-MAX karyotyping medium (ThermoFisher Scientific Inc.) was used for the CBMN assay. The same Gammacell 40 source used for the gene expression assay was used for irradiation. The dose rate was 0.70 Gy/min. All VacuStor and aliquoted samples were placed in the incubator at 37°C with 5% CO_2_. After 24 h of incubation, 250 μl of cell suspension from all samples were transferred into wells of a 96-well plate. Cytochalasin-B was added to the final concentration of 6 μg/ml and samples were cultured for another 30 h. After completion of culturing, cells were processed and analyzed as previously described [5].

## Results and discussions

### Integrated blood collector prototyping

The concept of packaging small individual parts using 3D printed enveloping layers and adaptors is straightforward, but several parts and mechanisms need to be fabricated and implemented in order to realize the functionality of the collector. To prototype the device, three enveloping layers were used to enclose the lancet and two capillary-needle-assemblies into one device, as shown in Fig 1. Press fitting was used to assemble the layers due to its simplicity, high strength and low cost [10]. The peripheral pillar/hole structures on the enveloping layers were pressed against each other and the friction force held the layers together. The two VacuStor tubes were housed inside a tube holder and the tube holder could be inserted into the device and slide along two guides printed on the middle enveloping layer for tube cap piercing. A holder safety was used to hold the tube holder together with the device during storage and prevent accidental cap piercing when in the LOCK position. The sliding action of the tube holder could be enabled by rotating the holder safety 90 degrees to the UNLOCK position. A capillary cap was used to prevent damage and contamination of the capillary tips during storage. All the parts were printed by 3D printing due to the iterative nature of the design and rapid prototyping capability of 3D printing. The insert of Fig 1 shows a finished integrated blood collector prototype.

To self-collect large volumes of fingerstick blood (100 to 200 μl) for the biodosimetry application, we designed the blood collector to sit on a table during collection so that the non-pricked hand of the subject can be used to conduct the milking action as needed. As shown in Fig 1, the tips of the capillaries were tilted upwards to aid the blood flow into the capillary. Different tilting angles up to 60 degrees were tested. We observed that a tilting angle as small as 3 degrees could allow sample to flow into the capillary quickly. For tilting angles larger than 30 degrees, the capillary force at the tip was not able to hold the sample against gravitational force and an air gap formed inside the capillary. In this study, a 5-degree tilting angle was used to prototype the collectors. Currently, the key components of the traditional collection kit (lancet, capillary, high-throughput storage tubes with liquid reagents) have been integrated into the collector. Additional items, such as alcohol wipes, gauze pad and BAND-AID, could also be integrated to the collector to prevent scattering during collection, e.g. attached to the sides of the collector. The hand warmer is expected to be used separately due to its current large size.

### VacuStor system characterization

The vacuum tube design for the integrated blood collector is not without challenges. The existing commercial vacuum tube systems are mainly designed for venipuncture with large tube sizes, and are not suitable for high-throughput bioassays where the storage tube volume is only ~ 1 ml or less. The shelf life of the small storage tube can be a concern due to vacuum loss from its high surface-to-volume ratio, such as in the recently reported microneedle blood extraction system [11]. Even though vacuum tubes have been used in clinical practice for a long time, no theoretical model has been published regarding the design of air evacuation for blood collection except some empirical observations, such as how blood draw volume varies with altitude [12]. In this paper, we use the ideal gas law and gas permeation theory to characterize two parameters that are important to the functionality and practical use of the VacuStor system, i.e. threshold vacuum pressure (P_th_) below which all the blood can be transferred from the capillary into the tube, and vacuum shelf life (T_sh_) of the tube due to vacuum loss.

#### Threshold vacuum pressure (P_th_)

**Fig 2:**
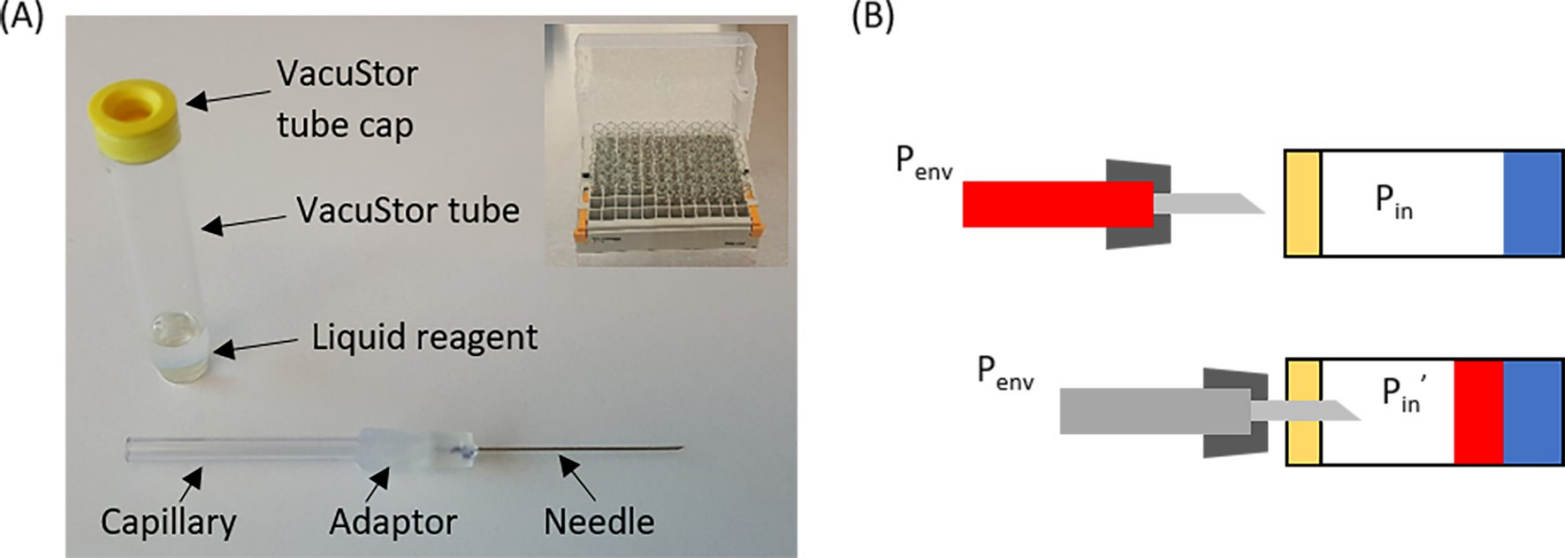
Threshold vacuum testing setup. (A) Image of a VacuStor tube and a capillary-needle assembly. The insert shows a 96-tube rack; (B) Schematics of VacuStor tube system before and immediately after transfer of all the blood.

Fig 2A shows an image of a nominal 1.0 ml glass VacuStor tube and a 100 μl capillary-needle-assembly used in this study. Fig 2B shows the schematics of a VacuStor tube before and immediately after transfer of all the blood from the capillary into the tube with P_in_ and P_in_’ as the respective tube pressures and P_env_ as the environmental pressure. To have a proper sample transfer, we have:

$${P_{in}}^{\prime }\leq P_{env}$$ (1)

At standard temperature and pressure where most blood collections are conducted, ideal gas is expected to be a good approximation for air. According to the ideal gas law, P_th_ can be deduced as:

$$P_{th}=\frac{V_{t}-V_{l}-V_{b}}{V_{t}-V_{l}}*P_{env}$$ (2)

where V_t_, V_l_ and V_b_ are the tube, liquid reagent and blood volumes respectively. Saturated water pressure at room temperature from aqueous reagent and needle capillary pressure are a few kPa or less, on the same order as the accuracy of the pressure gauge we used (±3% of 100 kPa), and are neglected here.

**Fig 3:**
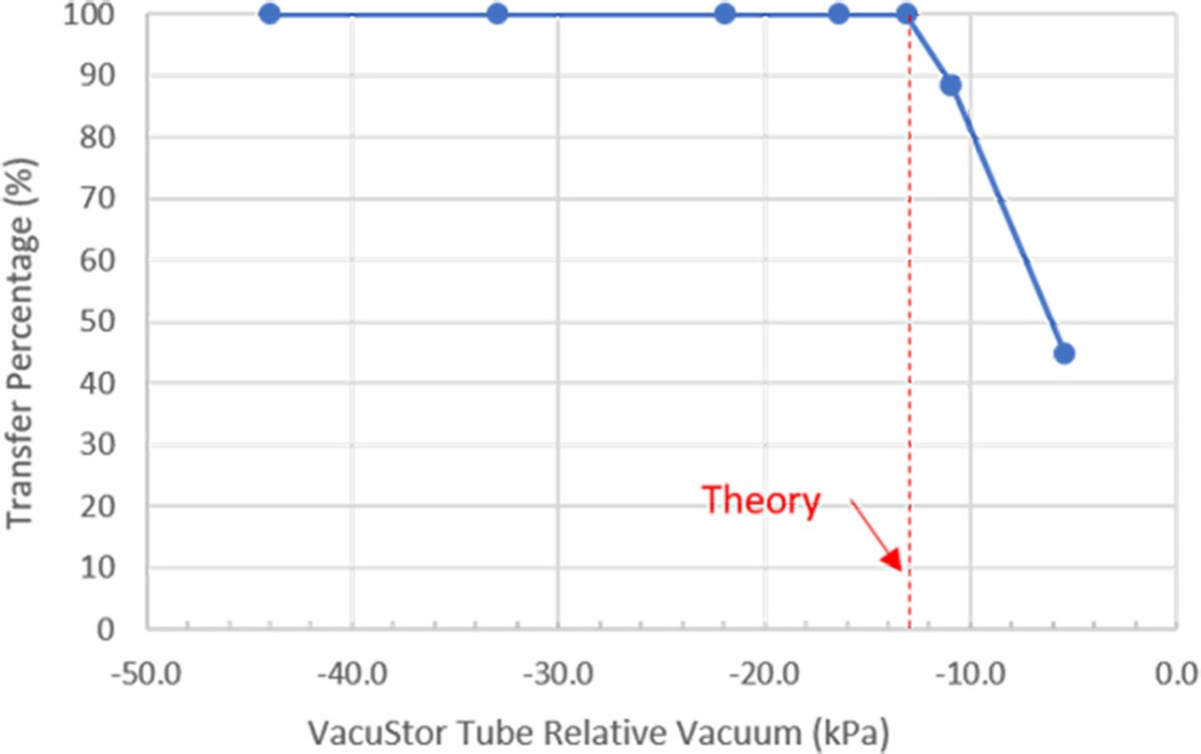
Threshold vacuum theoretical prediction and experimental result. Blue dots: experimental results of sample transfer percentage vs. VacuStor tube relative vacuum; red dashed line: relative threshold vacuum prediction by Eq (2).

It can be seen from Eq (2) that P_th_ depends on V_t_, V_l_, V_b_ and P_env_. To test if the ideal gas law can be used to design VacuStor P_th_ for small tube volume (1 ml or less), V_l_ and V_b_ volumes of 300 μl and 100 μl were used. V_t_ and P_env_ were measured experimentally to be 1042 μl and 96.8 kPa respectively. Fig 3 shows the experimental results of how the sample transfer percentage changed with the VacuStor tube vacuum relative to the environment (blue dots). The threshold vacuum was measured to be -13.1 kPa relative to P_env_. The calculated threshold relative vacuum (P_th_—P_env_) by Eq (2) was -13.0 kPa (red dashed line), indicating a good model of the ideal gas approximation in sub-milliliter regime after considering and accurately measuring all the small volumes involved in the process. The detailed volume correction and deduction of the VacuStor tube relative vacuum can be found in the 2^nd^ section of S1 File.

#### Vacuum shelf life (T_sh_)

Vacuum shelf life (T_sh_) of the VacuStor tube is related to the pressure increase from gas permeation of the rubber cap (the glass tube used in this study is considered non-gas-permeable). Using gas permeation theory [13] and considering gas composition of air, the normalized relative vacuum Δ*P*′(*t*) can be deduced as (See the 3^rd^ section of S1 File for details):

$${\Delta P}^{\prime }(t)=-\frac{P_{in,n}(t)+P_{in,o}(t)-P_{env}}{\left[(P_{in,n}(0)+P_{in,o}(0))-P_{env}\right]}=-\frac{{\Delta P}_{n}(t)+{\Delta P}_{o}(t)}{\Delta P(0)}=-0.785*e^{-\frac{t}{t_{n}}}-0.215*e^{-\frac{t}{t_{o}}}$$ (3)

Where Δ*P*_*i*_(*t*) = *P*_*in*,*i*_(*t*)−*P*_*env*,*i*_, Δ*P*(0) = Δ*P*_*n*_(0)+Δ*P*_*o*_(0), $t_{i}=\frac{V*\delta }{\eta_{i}*A*RT}$, V is the tube air space volume, δ, A and η_i_ are the cap thickness, area and permeability to gas i, i = n (nitrogen), o (oxygen), R is the gas constant, T is the absolute temperature. The negative sign of Δ*P*′(*t*) is to show that the pressure is below the environmental pressure.

**Fig 4:**
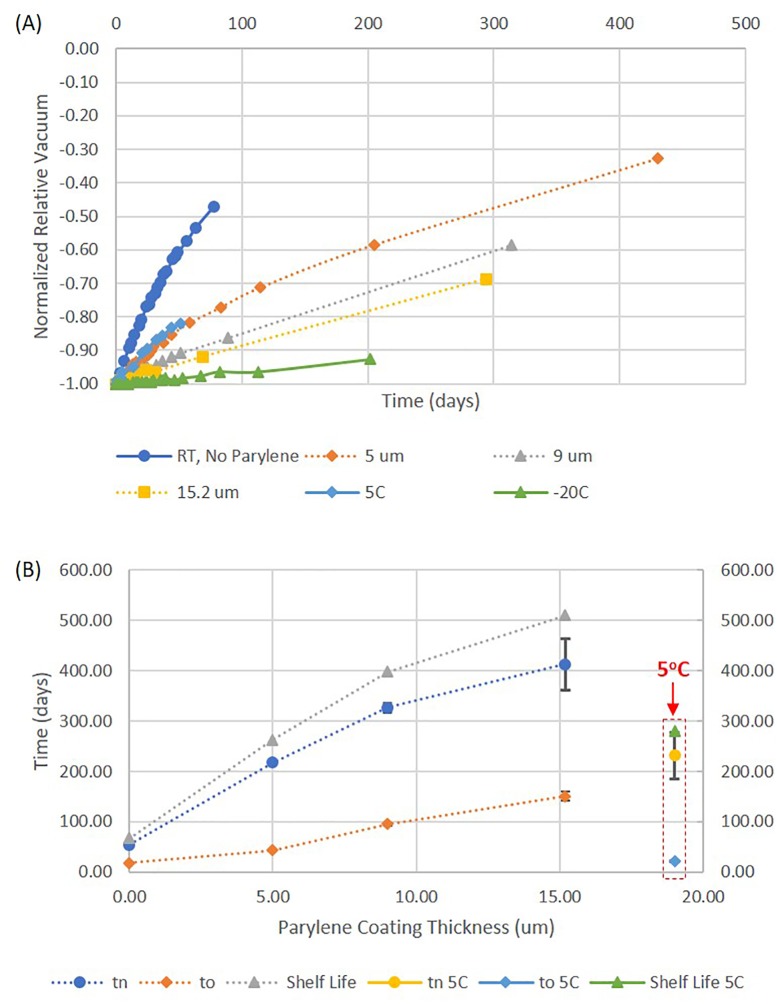
VacuStor tube vacuum shelf life study. (A) Change of normalized relative vacuum over time for VacuStor tubes under different conditions (Performance of one tube out of three replicates was plotted for each condition); (B) Means and standard deviations of nitrogen, oxygen leaking time constants for different Parylene coating thicknesses; the values for 5°C were also plotted for comparison. Three tubes were analyzed for each condition. Shelf lives were calculated using the mean values.

By drilling a hole at the bottom of the VacuStor tube, and gluing it to a vacuum gauge, the normalized relative vacuum pressures over time under multiple conditions were measured experimentally, as shown in Fig 4A. Because the vacuum gauge has an internal volume V_gauge_ of 1.45 ml that will increase the time constants, we denote the measured time constants as t_n_’ and t_o_’. It can be seen in Fig 4A that for a VacuStor tube at room temperature without any cap coating, the vacuum dropped to 47% in 77 days. By fitting the result using Eq (3) using Excel (from Microsoft), t_n_’ and t_o_’ were found to be 134.4 and 40.3 days respectively. The real t_n_ and t_o_ of the tube were calculated as 56.2 and 16.8 days. Assuming an initial vacuum Δ*P*(0) of -85 kPa and a threshold vacuum of -20 kPa, the shelf life of the VacuStor tube due to gas leakage was calculated by Eq (3) to be 67 days, too short to make the device commercially feasible.

For a practical shelf life of VacuStor tubes (e.g. 365 days), we consider the scaling law of the gas leaking time constant, which is on the same order of the shelf life. Eq (3) shows that t_i_ is proportional to V and δ, and inversely proportional to η_i_, A and T. Because miniaturization of the tube usually leads to increased surface to volume ratio (A/V) and smaller cap thickness δ, the shelf life of VacuStor tubes can be much shorter than that of larger evacuated tubes. To increase t_i_, η_i_ and T need to be reduced. Reduction of T is usually limited to only ~ 16% (from ~ 300K to 253K), which alone is not significant enough to extend the shelf life of the VacuStor tubes to ~ a year. However, lower T could reduce the permeability of the VacuStor tube cap due to the exponential Arrhenius rule [14], which is a possible way to extend the VacuStor tube shelf life to the desire value.

If T cannot be lowered, η_i_ of the cap needs to be lowered by other means. The current SepraSeal cap is made of a proprietary thermoplastic elastomer (TPE) from the manufacturer with no published data on gas permeability. However, we estimated the cap’s nitrogen and oxygen permeabilities using the gas leaking time constants fitted by our experiment (see the 3^rd^ section of S1 File). The values were 383 and 1129 cm^3^-mm/m^2^-day-atm respectively, and are listed in Table 1. Table 1 also listed other TPE permeability values reported in Ref. [14]. It shows that alternative TPEs with much lower permeability are available. Nonetheless, molding a cap using a new material is beyond the capability of our laboratory. However, there are other ways to improve barrier property of the existing cap, such as an additional barrier coating [14]. Thin metal or silicon oxide coating can lower permeability of plastics dramatically, but the processes are expensive and the coatings may crack for elastomer applications. Parylene C is another a common barrier coating [15] with permeability data much smaller than TPEs (Ref. 14, Table 1). It is also a polymer material that is suitable for elastic cap application. In this project, we tested Parylene C coating to extend the VacuStor tube vacuum shelf life.

**Table 1:** Nitrogen and oxygen permeability data for SepraSeal cap and other relevant plastic materials; unavailable data are labeled “n/a”.

Materials	Gas Permeability (cm^3^-mm/m^2^-day-atm)	
	Nitrogen	Oxygen
SepraSeal Cap	383	1129
Thermoplastic Elastomers (TPEs)	9–1116	34–1620
Parylene C	0.4	2.8
ARY 3-mil Vacuum Bag	n/a	3.66

Fig 4A shows our experimental data of how the normalized relative vacuum changed for VacuStor tubes stored at 5°C and -20°C, as well as stored at room temperature with 5, 9, 15.2 μm Parylene C coating on the cap. It can be seen that, indeed, Parylene coatings reduced the gas leakage significantly; refrigerator temperature (5°C) also reduced the gas leakage, similar to a 5μm Parylene coating; freezer temperature (-20°C) lowered gas leakage the most.

Fig 4B shows the mean values and standard deviations of fitted nitrogen, oxygen leaking time constants for three tubes at each condition for different Parylene C thicknesses. The variation is larger for thicker coatings because of the smaller vacuum loss within the experimental time frame due to reduced permeability. The time constants also increase linearly with polymer thickness except for t_n_ at 15.2 μm. This is consistent with our theoretical analysis of gas leakage time constant for a two-layered cap (see Eq (S7) in S1 File). The reduction of the t_n_ at 15.2 μm could come from degraded cap sealing due to stress from the thicker polymer. It should also be noted that because the gases besides nitrogen occupy only 22% of air, the threshold vacuum may not be reached even if all the other gases reached equilibrium with the environment. That means the shelf life of VacuStor will mostly be determined by the leakage of the nitrogen gas, i.e. t_n_. In Fig 4B (gray line), the shelf lives for different Parylene thicknesses were calculated and plotted using mean values of t_n_, t_o_ and the previously assumed initial and threshold relative vacuums. The shelf life showed a linear increase with Parylene thickness with a reduction at 15.2 μm, similar to the trend of t_n_, but not t_o_ which shows a linear increase for all Parylene thicknesses, consistent with our analysis. It should also be noted that a 9 μm Parylene coating has an expected shelf life of 389 days, long enough for practical applications.

Fig 4B also shows the mean values and standard deviations of fitted nitrogen, oxygen leaking time constants for three tubes without Parylene coating at 5°C. For -20°C, we cannot fit t_n_ and t_o_ reliably because the limited vacuum loss due to extremely slow gas leakage and an interruption of the experiment at day 202. Using the Arrhenius Equation and the leaking time constatns at room temperature (300 K) and 5°C (278 K), we calculated the activation energy of the nitrogen permeability for the SepraSeal caps to be 43.3 kJ/mol. If this activation energy still applies at -20°C, it will give a nitrogen leaking time constant of 1619 days, which makes low temperature storage a viable way to extend the VacuStor tube vacuum shelf life to be over a year and is also consistent with the very low gas leakage we observed experimentally. The activation energy for oxygen permeability was not calculated because the leaking time constants did not show significant changes between room temperature and 5°C. Detailed temperature analysis of the SepraSeal cap gas permeabilities can be found in the 3^rd^ section of S1 File.

**Fig 5:**
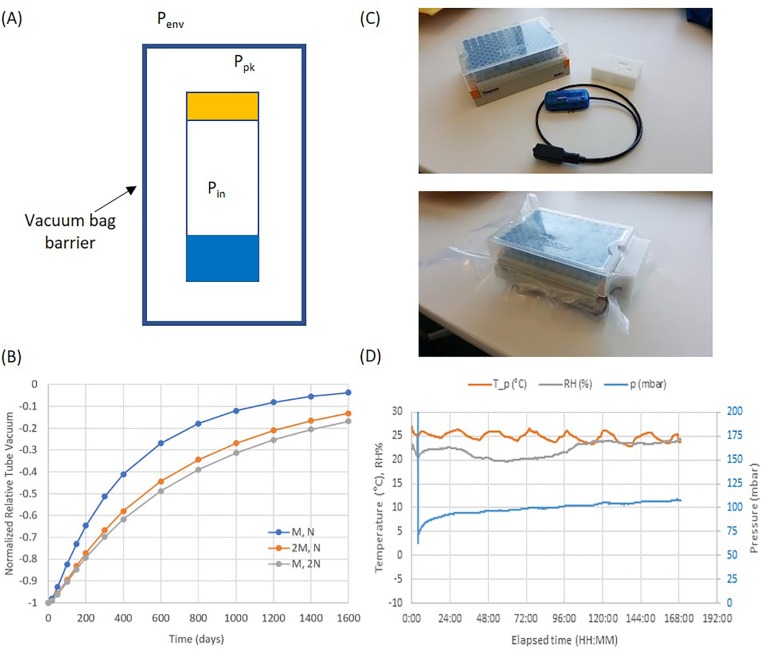
Packaging of VacuStor tubes inside a container by vacuum bag sealing. (A) Schematics; (B) Theoretical glass VacuStor tube vacuum decay for different M and N values; (C) Experimental setup showing a 96-tube rack with a data logger being sealed within a vacuum bag and a plastic fixture to prevent data logger buttons being pressed by the vacuum bag after sealing; (D) logger data showing how the container pressure P_pk_ changed after sealing for 7 days.

Due to the difference of storage conditions between gene expression and CBMN reagents (RNA stabilization solution stored at room temperature and cell culture medium stored at -20°C), it may be necessary that the VacuStor tubes be stored separately from the collector and pre-installed into the collector just before use. For this scenario, a process to extend vacuum shelf life by sealing VacuStor tubes inside a container using low cost vacuum bag sealing was tested, as shown in Fig 5A. In this way, the tube vacuum is protected by both the tube barrier and the vacuum bag barrier. The additional volume provided by the container (i.e. the space between the vacuum bag and the VacuStor tube) can also slow down the air leakage into the tube. Vacuum bag sealing is a much lower cost process than Parylene coating. Current commercial vacuum bags, such as a 3-mil vacuum bag from ARY (Overland Park, KS) used in this study, can also have similar gas permeability to Parylene C (Table 1, from the manufacturer).

To study how the barriers and the additional container volume affect the VacuStor tube vacuum, we were able to deduce a mathematical solution for the normalized relative tube vacuum (see details in the 3^rd^ section of S1 File). The tube vacuum decay depends on the gas leaking time constants of the tube plus two more factors, M (ratio of the additional container volume to the VacuStor tube headspace volume) and N (ratio of the vacuum bag gas leaking time constant to the VacuStor tube gas leaking time constant). To get an idea of how good the vacuum bag sealing is, we estimated M and N for the scenario in which 96 empty glass VacuStor tubes were sealed by the 3-mil ARY vacuum bag inside their rack holder. M and N were estimated to be 2.755 and 2.417 (Nitrogen permeability of the vacuum bag is unknown, and the N value was assumed to be the same as that for oxygen). Fig 5B shows how the normalized relative tube vacuum decayed over time. Using the previously assumed initial and threshold vacuums, the VacuStor tube vacuum shelf life was found to be 666 days. This indicates vacuum bag container sealing is a promising way for long time VacuStor tube storage. Furthermore, doubling N (i.e. the vacuum bag barrier) almost doubled the vacuum shelf life (1274 days). Doubling M (i.e. the additional container volume) also significantly increased the vacuum shelf life (1110 days), but not as much as doubling N (Fig 5B). Detailed optimization of M and N for vacuum bag sealing of VacuStor tubes are beyond the scope of this manuscript and will be discussed elsewhere.

For experimental characterization, it is hard to measure vacuum pressure inside the VacuStor tube due to its limited space. However, we did measure the container pressure P_pk_ for vacuum bag sealed 96-tube rack with a limited time span (7 days). Fig 5C shows a rack of 96 polypropylene 1.4 ml VacuStor tubes with a data logger to collect data for the container space, which were then sealed within the 3-mil ARY vacuum bag. The polypropylene tubes were used because we believe the measured P_pk_ would be closer to the tube vacuum pressure due to its higher gas permeability than the glass tubes. A white plastic fixture was also machined to prevent direct pressure of the vacuum bag on the logger push buttons. Fig 5D shows how the humidity, temperature and pressure changed over a time of 7 days. The measurement time is limited by the battery life of the logger. It can be seen that P_pk_ had an initial value of ~ 62 mbar with a quick increase of ~ 31 mbar for day 1, possibly due to initial outgassing of sealed materials. Then P_pk_ increased ~ 2.3 mbar/day for the remaining 6 days. At this rate, it would take 289 days for P_pk_ to increase to the threshold vacuum. Considering the leakage rate will drop significantly when P_pk_ approaches the environmental pressure, the time it will take for P_pk_ to reach the threshold vacuum would be much longer than 289 days. Because P_pk_ is always higher than the tube pressure, the VacuStor tube vacuum shelf life would be even longer. The fact of being able to extend vacuum shelf life of polypropylene tubes, which have higher permeability than glass tubes, to ~ years demonstrated experimentally the feasibility of vacuum bag sealing for long term VacuStor tube storage.

The current VacuStor vacuum analyses are based on fixed environmental pressure P_env_ at ~ sea level. It is possible for VacuStor tube to be used at higher elevation, where environmental pressure P_env_’ is lower than P_env_. From Eq (2), this means P_th_’ < P_th_, i.e. a better tube vacuum is needed to transfer the sample at higher elevation. This will reduce the vacuum shelf life of the tube. However, multiple ways to extend the vacuum shelf life discussed earlier should be able to address this issue, such as low temperature storage, better barrier materials, or additional container space.

### Self-collection testing

Fingerstick blood self-collection has been used at home for diabetes management and research. But the collection volume was low (1 to 15 μl) so that finger milking action was not necessary [16]. Our literature search has not revealed any study of self-collection of > 100 μl blood from fingerstick, although such practice may exist with less demands than the biodosimetry assays (e.g. liquid reagent storage, blood metering etc.). To test the self-collection of our integrated blood collector, we want to first make sure that the desired volume of blood can be generated from a single prick from the collector. We conducted preliminary testing of the blood volume that could be generated by our selected lancet (BD Blue Microtainer contact-activated lancet) using a traditional collection process (see the 4^th^ section of S1 File). Without using a hand warmer, 3 out 7 collections reached 200 μl vs. 6 out of 7 collections reached 200 μl using a hand warmer. To minimize the chance that a single prick may not generate enough blood for the collector, we tested the integrated blood collector with a collection volume target of 100 μl (two 50 μl capillaries) with a hand warmer.

Ten healthy donors ages 20 to 51 without any fingerstick blood collection experience were recruited. They were asked to self-collect the blood samples into VacuStor tubes after watching an instructional video (see S2 File). The instructional video was made because the collection process still involves multiple steps and we considered that video education would be more effective than written instructions, as reported previously [17]. Table 2 shows the results of the test, including donor number, capillary size and collected blood volume. The collected blood volume into the VacuStor tubes was measured by weighing the tubes before and after collection and converting the weight to volume using a blood density of 1.060 g/ml. All 10 donors were able to successfully collect blood using the collector. Among the 20 capillary-needle-assemblies from the 10 devices, 18 were able to collect blood and transfer the blood into the VacuStor tubes by vacuum. The blood volume collected ranged from 43.1 to 50.9 μl, with mean±SD of 47.1±2.1 μl. No significant blood volume was left in the capillaries after transfer. We attribute the blood volume variation to our fabrication process where the capillary was manually cut out from a commercial product. For two capillary-needle assemblies, the capillaries were not able to collect the blood, possibly due to clogging of the assemblies during the fabrication process. After introducing a quality control measure to identify clogged capillary-needle assemblies by passing through compressed air, this phenomenon has not been observed during the later biodosimetry study.

**Table 2:** Results of the integrated blood collector self-collection testing.

Donor#	Capillaries	Before (g)	After (g)	Blood Volume (μl)
1	50 μl x 2	1.8881	1.9348	**44.1**
		1.8722	1.9190	**44.2**
2	50 μl x 2	1.87213	1.92251	**47.5**
		1.86793	1.92090	**50.0**
3	50 μl x 2	1.87618	1.92787	**48.8**
		1.87569	1.92646	**47.9**
4	50 μl x 2	1.90384	1.95384	**47.2**
		1.89575	1.94385	**45.4**
5	50 μl x 2	1.89249	1.94239	**47.1**
		1.89922	1.9504	**48.3**
6	50 μl x 2	1.89658	1.94827	**48.8**
		1.90226	1.95273	**47.6**
7	50 μl x 2	1.86968	1.91537	**43.1**
		1.88129	1.93077	**46.7**
8	50 μl x 2	1.88586	failed	**failed **
		1.88879	1.9363	**44.8**
9	50 μl x 2	1.91799	failed	** failed**
		1.85468	1.90059	**43.3**
10	50 μl x 2	1.89444	1.94838	**50.9**
		1.88652	1.93616	**46.8**

Even though all donors successfully collected blood themselves, it was not without issues. Two out of ten donors forgot to disinfect their fingers before prick. Two donors also had blood drip to the back of their hands due to upward finger positions. These will be further improved through refined instructions and possible device design. Even with these issues, however, no infection was reported from the donors.

### Gene expression and CBMN biodosimetry assays

In order to further validate the integrated blood collector for biodosimetry applications, we compared samples collected by the device to those by traditional method using both gene expression and CBMN assays.

#### Gene expression assay

**Fig 6:**
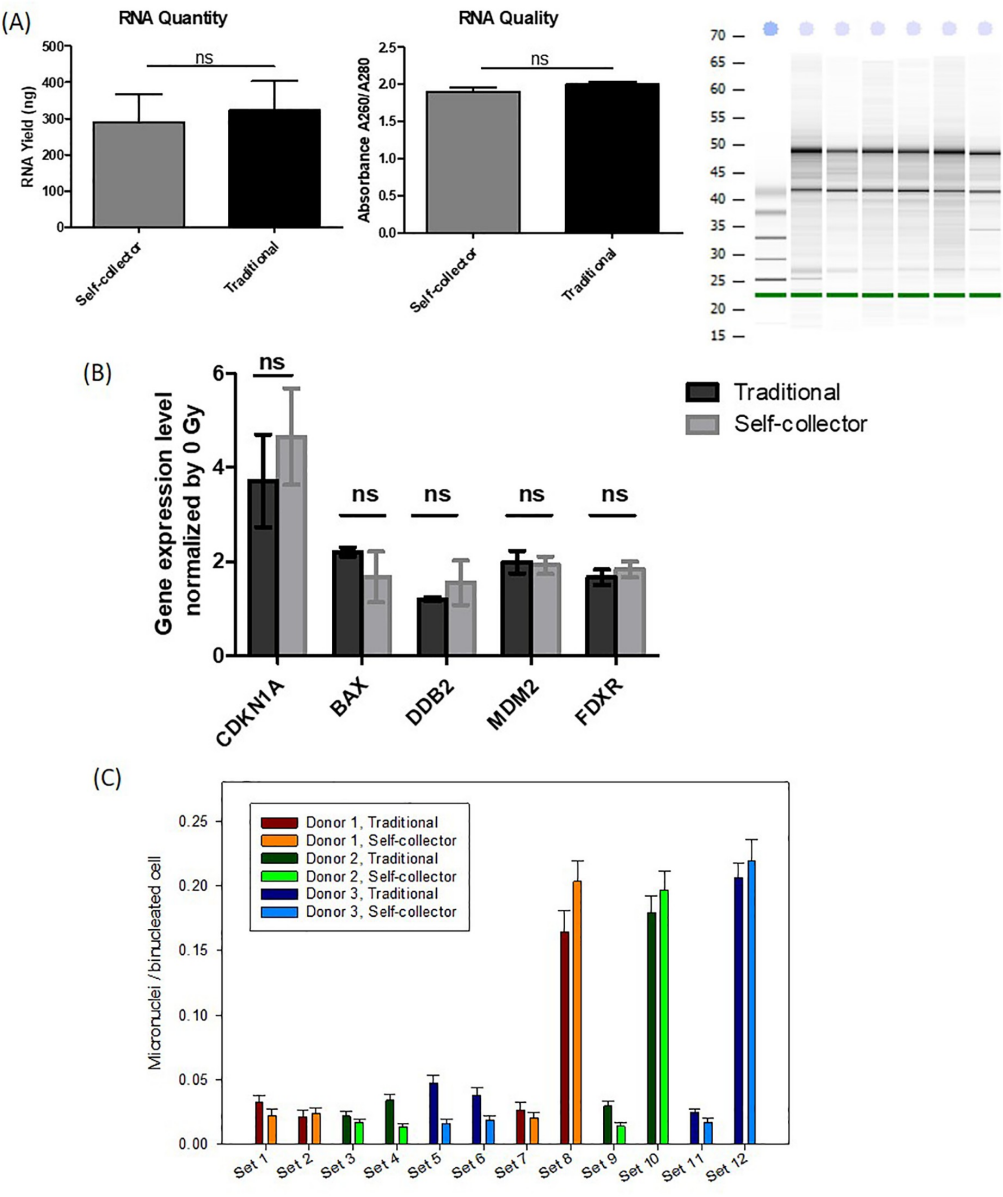
Characterization of self-collected samples for gene expression and cytogenetic biodosimetry assays. (A) Comparison of quantity (left) and quality (middle) of RNA isolated from blood using traditional method and the self-collector, the electropherogram (right) shows integrity of RNA by the self-collectors (RINs = 7.0–8.5, comparable to traditional method); (B) change of gene expression at 3 Gy relative to 0 Gy for real-time qRT-PCR (NS = not significant); (C) Micronuclei per binucleated cell for samples collected by traditional method or the integrated self-collector. The numbers are below 0.05 for all non-irradiated samples, and no significant difference between traditional and self-collector for the irradiated samples.

For gene expression assay, quality and quantity of isolated RNA were first compared using non-irradiated blood. Blood (50 μl) was collected into microcentrifuge tubes containing RNA stabilization solution by traditional method from three healthy donors. Another 50 μl of blood was collected into VacuStor tubes containing the same reagent by the integrated blood collectors from the same donors at the same time. RNA was isolated and the results are shown in Fig 6A. No significant difference (P>0.05) was found between the two methods in terms of the quantity or quality of the isolated RNA. Some 300–400 ng of total RNA was isolated from 50μl of blood using the device, which is comparable to that by traditional fingerpick collection method. The quality of RNA was also characterized by absorbance ratio at 260 and 280 nm and was found to be comparable to that by the traditional method. Fig 6A also shows an electropherogram of some of the RNAs isolated from samples collected by the devices; the RINs (RNA integrity number) of the RNAs as determined by an Agilent Bioanalyzer were found to be between 7.0–8.5, which is of acceptable quality for most downstream applications.

Besides the RNA quantity and quality, an experiment was also conducted to see if sample collection by the integrated blood collector would alter radiation-induced gene expression comparing to traditional method using a real-time qRT-PCR assay. Five radiosensitive genes (*CDKN1A*, *BAX*, *DDB2*, *MDM2 and FDXR*) reported in the literature [18] were selected for the experiment. Because it is complicated to irradiate blood inside the collector, venous blood was collected from three healthy donors into two sample tubes. One tube was irradiated to a dose of 3 Gy, and the other tube was used as a sham-irradiated control. After allowing time for gene expression changes to occur, the irradiated and control bloods were collected into the VacuStor tubes containing the RNA stabilization solution through the device, or pipetted into microcentrifuge tubes containing the same solution. The gene expression levels of the 3 Gy samples relative to those of unirradiated controls were measured using both the traditional method and through the device. The housekeeping gene *ACTB* was used as the normalization control. We were able to detect expression of all 5 genes using the collector. The gene expression levels and radiation response trends measured using the device were similar to the measurements made by a traditional pipetting process (no significant difference in expression at P>0.05, see the 5^th^ section of S1 File for detailed data), indicating that blood collection by the collector can be used for gene expression analysis (Fig 6B).

Finally, a gene expression comparison between the traditional method and the integrated blood collector using a ligation based assay chemistry (DxDirect®, DxTerity Diagnostics, CA) was also conducted, and the gene expressions were also found to be “not significant” between the two methods (see the 5^th^ section of S1 File), indicating a broader application of the device for different assay chemistries.

#### Cytogenetic CBMN assay

We first compared micronuclei level for non-irradiated fingerstick samples. For traditional fingerstick collection, 150–200 μl of blood per donor from three healthy donors were collected into heparinized tubes. Then 25 μl aliquot was dispensed into the 1 ml glass Matrix tube containing 225 μl of culture medium for the CBMN assay. For the integrated blood collector, six 50 μl blood samples (two samples per donor from the same three donors) were also collected at the same time by the collector into VacuStor tubes containing 550 μl culture medium. The level of micronuclei per binucleated cell was measured for all the non-irradiated samples, as shown in Fig 6C (sets 1–6). The numbers were all below 0.05, which is an acceptable background (5).

For irradiated samples, venous blood was collected from the donors for the same reason as mentioned in the gene expression assay. The blood was then split in half, and irradiated at 0 and 3 Gy respectively by gamma-rays. Then the irradiated samples and the controls either went through the collector to be collected into VacuStor tubes, or were dispensed in aliquot into the 1 ml matrix tubes as described for the non-irradiated fingerstick sample experiment above. The number of micronuclei per binucleated cell was assayed by CBMN, as shown in Fig 6C (sets 7–12). The 0 Gy samples prepared with or without the collector still showed the micronuclei level to be below 0.05. The 3 Gy samples showed no significant difference in the micronuclei level when they were prepared with or without use of the collector for all 3 donors (P>0.05). These results indicate that the integrated blood collector can be used for blood collection for cytogenetic analysis to estimate radiation doses.

## Conclusion

We conceived and prototyped an integrated fingerstick blood collector with liquid reagent storage capability for high-throughput sample self-collection and pre-processing suitable for use with large-scale radiation biodosimetry. A critical sub-system of the collector, the VacuStor system, has been characterized and a methodology for analyzing evacuated tubes for blood collection has been developed. We have shown that the threshold vacuum of the VacuStor tube can be deduced by the ideal gas approximation. Through gas permeation theory, different ways to extend the shelf life of the VacuStor tube over a year were developed, including low temperature storage, Parylene barrier coating and container vacuum bag sealing. The collector has been successfully tested for self-collection, and the collected blood was also successfully demonstrated for gene expression and cytogenetic biodosimetry assays. Besides radiation countermeasures, the device could be used for other applications where blood may need to be self-collected by individuals (e.g. at home or in a disaster theater) with liquid reagent pre-processing and sent to a central laboratory for analysis. The vacuum tube methodology developed here could also be used to guide the development of other miniature vacuum actuated devices [19].

## Supporting information

S1 File Supplementary materials. (PDF)

S2 File Educational video. The educational video for conducting self-collection of blood sample using the developed blood collector.
(3GP)
