# Supplementary material for: Development of an integrated fingerstick blood self-collection device for radiation countermeasures
Source: PLoS One. 2019 Oct 16;14(10):e0222951. doi: 10.1371/journal.pone.0222951 (PMC6795524; doi:10.1371/journal.pone.0222951)
Supplement: S1 File — (PDF) [file pone.0222951.s001.pdf]

Development of an integrated fingerstick blood self-collection device for radiation countermeasures

Jian Gu[1*], Alan Norquist[1], Carla Brooks[1], Mikhail Repin[2], Sanjay Mukherjee[2], Jerome Lacombe[1], Jianing Yang[1], David J. Brenner[2], Sally Amundson[2] and Frederic Zenhausern[1*]

[1]Center for Applied NanoBioscience and Medicine, The University of Arizona, College of Medicine, Phoenix, AZ 85004; [2]Center for Radiological Research, Columbia University, Vagelos College of Physicians and Surgeons, New York, NY 10032

Supplementary Materials:

S1. VacuStor tube formation:

Fig S1A shows the fixture that fabricates VacuStor tubes one at a time. The fixture was made with a plastic tube with two rubber stoppers sealing at each end. To seal a VacuStor tube, the Matrix storage tube with liquid reagent seated on the bottom stopper, the tube cap seated on a plastic rod through the top stopper, and the tube can be sealed by pushing the cap to the opening of the tube. A vacuum port was used to pump vacuum. A vacuum gauge (GZ43-K-01, SMC Corporation) was used to read the vacuum relative to the environmental pressure.

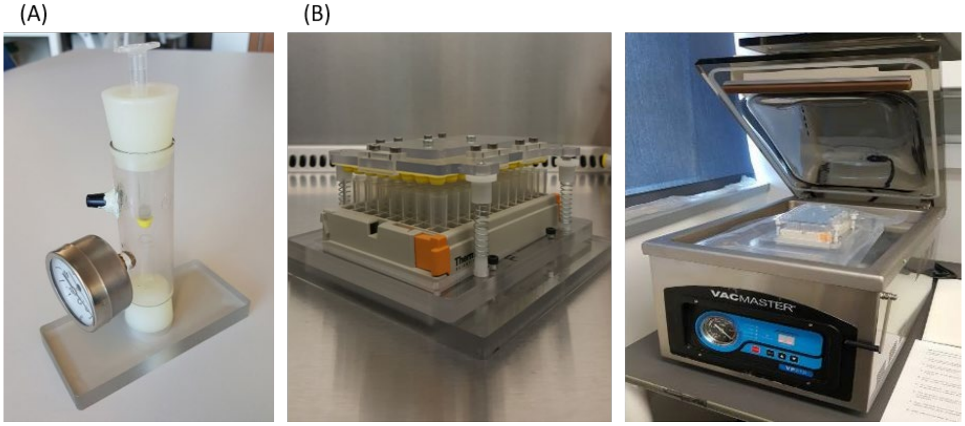

Fig S1 Setups for sealing VacuStor tubes. (A) vacuum fixture to form VacuStor tubes one at a time; (B) the fixture (left) and the chamber vacuum sealer (right) to seal 96 VacuStor tubes simultaneously.

Fig S1B shows the setup to seal 96 VacuStor tubes in a rack at the same time. A fixture was made to have all the caps on a mat suspended over the open tubes. The fixture was put inside a vacuum sealing bag and sealed by the chamber vacuum sealer (VacMaster VP210). After vacuuming and bag sealing, the chamber was opened, and all the tube caps were pressed onto the tube openings by atmosphere pressure to seal all the VacuStor tubes.

S2. VacuStor Threshold Vacuum Testing:

VacuStor tubes were sealed with different vacuum gauge readings (i.e. P_{fix} - P_{env}) of -49, -39, -29, -24, -21, -19 and -14 kPa, where P_{env} was measured to be 96.8 kPa and P_{fix} was the vacuum pressure of the fixture during tube sealing. At the beginning of the tube sealing, the tube pressure was the same as P_{fix}. However, there was a tube volume reduction (V_c) during VacuStor tube fabrication between initial tube sealing and fully seated cap. Vc was measured by the geometries of the cap to be 77 µl. This caused increase of the tube pressure. Using the ideal gas law, we have $P_{in} = \frac{(V_t-V_l)+V_c}{(V_t-V_l)} * P_{fix}$, where V_t was the tube volume after cap seating (measured as 1042 µl) and P_{in} was the tube vacuum pressure with fully seated cap. The relative tube vacuums ($P_{in} - P_{env}$) were corrected to be -44, -33, -22, -16.4, -13.1, -10.9 and -5.4 kPa.

S3. VacuStor tube vacuum loss calculation

Simple VacuStor tube model:

We approximate the gas permeation by Fick's first law:

$$j = \frac{D(C_{env}-C_{in})}{\delta} = \frac{DS(P_{env}-P_{in})}{\delta} = \frac{\eta(P_{env}-P_{in})}{\delta} \quad \text{(S1)}$$

where j is the flux density, D is the diffusion coefficient, C_{env} and C_{in} are the gas concentrations of the environment and inside the tube respectively, δ is the cap thickness, P_{env} and P_{in} are the outside and tube pressures, S=C/P is the sorption equilibrium parameter, and η=S*D is the permeability. Then the mass transfer flux rate:

$$I = j * A = \eta * (P_{env} - P_{in}) * A/\delta \quad \text{(S2)}$$

where A is the area of the cap. From the ideal gas law, we also have:

$$I = \frac{dN}{dt} = \frac{V}{RT}\frac{dP_{in}}{dt} \quad \text{(S3)}$$

where N is the amount of gas in moles, V is the tube air volume, R is the gas constant and T is the absolute temperature. From Eq. (S2) and (S3), the internal gas pressure change over time can be deduced as:

$$\Delta P(t) = \Delta P(0) * e^{-\frac{t}{t_{lk}}}, \text{ or } \Delta P'(t) = -\frac{\Delta P(t)}{\Delta P(0)} = -e^{-\frac{t}{t_{lk}}} \quad \text{(S4)}$$

where $t_{lk} = (V * \delta)/(\eta * A * RT)$ is the leaking time constant, $\Delta P(t) = P_{in}(t) - P_{env}$ is the relative vacuum, $\Delta P(0) = P_{in}(0) - P_{env}$ is the initial relative vacuum, and $\Delta P'(t)$ is the normalized relative vacuum (the negative sign is to show the pressure is below the environmental pressure).

Air: gas mixture

One thing to be noticed is that air is a mixture of multiple gases that their permeability for the rubber cap can be different. With the ideal gas approximation, all the gas molecules interact with each other only through elastic collisions, and each gas can be treated independently. For the tube pressure increase through permeation, we will consider only the two most abundant gases in the air, i.e. nitrogen (78%) and oxygen (21%), and the remaining 1% (mainly argon) will be treated as 0.5% of nitrogen and 0.5% of oxygen to simplify the situation. If we assume nitrogen and oxygen are pumped out of the tube at the same rate so that they have the same percentage in the evacuated tube at time zero as in the air, then:

$$\Delta P_n(0) = P_{in,n}(0) - P_{env,n} = 0.785[P_{in}(0) - P_{env}] = 0.785\Delta P(0)$$

$$\Delta P_o(0) = P_{in,o}(0) - P_{env,o} = 0.215[P_{in}(0) - P_{env}] = 0.215\Delta P(0)$$

and the total normalized relative vacuum:

$$\Delta P'(t) = -\frac{P_{in,n}(t) + P_{in,o}(t) - P_{env}}{\left[\left(P_{in,n}(0) + P_{in,o}(0)\right) - P_{env}\right]} = -0.785\frac{\Delta P_n(t)}{\Delta P_n(0)} - 0.215\frac{\Delta P_o(t)}{\Delta P_o(0)} = 0.785 * e^{-\frac{t}{t_n}} - 0.215 * e^{-\frac{t}{t_o}}$$

Where $t_i = \frac{V * \delta}{\eta_i * A * RT}$ (i=n, o) (S5)

SepraSeal cap permeability deduction from gas leaking time constant:

If t_i are known, gas permeabilities can be estimated by changing Eq. (S5) to be

$$\eta_i = \frac{V*\delta}{A*t_i*RT} \text{ (i=n, o)} \quad \text{with a unit of "mole-mm/m}^2\text{-day-atm"} \tag{S5'}$$

If a unit of "cm^3-mm/m^2-day-atm" is wanted for permeability, "mole" can be converted to "standard cm^3" by multiplying a conversion factor of standard gas volume V_{stp}=22.4*10^3 cm^3/mole. Then the new permeability η_i' can be expressed as:

$$\eta_i' = \eta_i * V_{stp} = \frac{V*\delta}{A*t_i*RT} * V_{stp} = \frac{V*\delta}{A*t_i*RT} * \frac{RT_{stp}}{P_{stp}} = \frac{V*\delta}{A*t_i*P_{stp}} * \frac{T_{stp}}{T} \tag{S5''}$$

where P_{stp} and T_{stp} are standard pressure (1 atm) and temperature (273 K).

To estimate the SepraSeal cap permeability, we used the following parameter values: V of tube 1.042 ml, δ of cap 1.1 mm, A of cap 50 mm^2, T 300 K, and the average fitted t_n and t_o from our experiment (mean$\pm$std of 54.46$\pm$2.32 from 49.20, 57.99, 56.19 days, and mean$\pm$std of 18.47$\pm$0.84 from 18.41, 20.18, 16.84 days respectively). The average nitrogen and oxygen permeabilities were estimated to be 383 and 1129 cm^3-mm/m^2-day-atm.

Two-layered cap:

For a two-layered cap, from Eq. (S2) we have:

$$(P_{env} - P_{int}) = \frac{I}{A} * \frac{\delta_1}{\eta_1} \text{ and } (P_{int} - P_{in}) = \frac{I}{A} * \frac{\delta_2}{\eta_2}, \text{ so that } (P_{env} - P_{in}) = \frac{I}{A} * (\frac{\delta_1}{\eta_1} + \frac{\delta_2}{\eta_2}) \tag{S6}$$

Similar to Eq. (S4), we have:

$$\Delta P'(t) = -\frac{\Delta P(t)}{\Delta P(0)} = -e^{-\frac{t}{t_{lk}}}, \text{ where } t_{lk} = t_{lk,1} + t_{lk,2} = \frac{V*\delta_1}{\eta_1*A*RT} + \frac{V*\delta_2}{\eta_2*A*RT} \tag{S7}$$

Temperature dependence of SepraSeal cap gas permeabilities by the Arrhenius Equation

Temperature dependence of gas permeabilities of plastic usually follow the Arrhenius equation as described below:

$$\eta(T) = \eta_0 \exp(\frac{-\Delta E_\eta}{RT}) \quad \text{(S8)}$$

where ΔE_η and η_0 are characteristics of a particular material and permeant pair. ΔE_η is also called activation energy with a unit of kJ/mol. If permeabilities of a material at temperatures $T_1 > T_2$ are known, the activation energy can be calculated by Eqs. (S8, S5") as:

$$\Delta E_\eta = ln\left[\frac{\eta(T_1)}{\eta(T_2)}\right] / \left(\frac{1}{RT_2} - \frac{1}{RT_1}\right) = ln\left[\frac{t_2 * T_2}{t_1 * T_1}\right] / \left(\frac{1}{RT_2} - \frac{1}{RT_1}\right) \quad \text{(S9)}$$

where t_1 and t_2 are the gas leaking time constants of the material at temperature T_1 and T_2.

Using the gas leaking time constants measured at room temperature (300 K, 54.46 and 18.47 days respectively for nitrogen and oxygen) and 5°C (278 K, mean$\pm$std of 231.85$\pm$46.09 from 191.24, 337.36, 166.95 and mean$\pm$std of 21.08$\pm$0.89 from 21.93, 22.27, 21.08 days respectively for nitrogen and oxygen), the activation energy of the SepraSeal cap for nitrogen permeability was calculated to be 43.3 kJ/mol. This is consistent with the activation energy reported for other plastic materials (Ref. 14). If we assume this activation energy also applies at -20°C, the nitrogen leaking time constant of the SepraSeal cap for -20°C can be calculated by Eq. (S9) as 1619 days using the time constant at either room temperature or 5°C. The activation energy for oxygen permeability was not calculated because the oxygen leaking time constants were statically the same for the two temperatures (P-value of 0.138 for two-tailed unequal-variance t-Test).

Vacuum bag sealing of VacuStor tubes inside a container

For vacuum bag sealing of VacuStor tubes, Fig 5A shows a schematic of the storage concept: the VacuStor tubes will be inside a larger container and the container is sealed by a vacuum bag barrier. To deduce the vacuum loss due to gas permeation for the VacuStor tube headspace, we have:

$$\frac{V_{t,h}}{RT}\frac{dP_{in}}{dt} = \frac{dN_0}{dt} = I_0 = \frac{\eta_0}{\delta_0} * A_0\left(P_{pk} - P_{in}\right) \quad \text{(S10)}$$

where N_0 and $V_{t,h}$ are the gas amount and volume of the tube head space, A_0, δ_0 and η_0 are the area, thickness and gas permeability of the tube barrier, P_{pk} is the pressure of the package container. For the package container space, we have:

$$\frac{V_{pk}}{RT}\frac{dP_{pk}}{dt} = \frac{dN_{pk}}{dt} = I_1 - I_0 = \frac{\eta_1}{\delta_1} * A_1\left(P_{env} - P_{pk}\right) - \frac{\eta_0}{\delta_0} * A_0\left(P_{pk} - P_{in}\right) \quad \text{(S11)}$$

where N_{pk} and V_{pk} are the gas amount and volume of the package container space excluding the VacuStor tubes, A_1, δ_1 and η_1 are the area, thickness and gas permeability of the container barrier.

Rearrange Eq. (S10-11), we have:

$$\frac{dP_{in}}{dt} = \frac{\eta_0 * A_0 * RT}{\delta_0 * V_{t,h}}\left(P_{pk} - P_{in}\right) = \alpha_0 P_{in} + \beta_0 P_{pk},\ where\ \alpha_0 = -\beta_0\ and\ \beta_0 = \frac{\eta_0 * A_0 * RT}{\delta_0 * V_{t,h}} \quad \text{(S12)}$$

$$\frac{dP_{pk}}{dt} = \frac{\eta_1 * A_1 * RT}{\delta_1 * V_{pk}}\left(P_{env} - P_{pk}\right) - \frac{\eta_0 * A_0 * RT}{\delta_0 * V_{pk}}\left(P_{pk} - P_{in}\right) = \alpha_1 P_{in} + \beta_1 P_{pk} + CP_{env} \quad \text{(S13)}$$

where $\alpha_1 = \frac{\eta_0 * A_0 * RT}{\delta_0 * V_{pk}} = \frac{V_{t,h}}{V_{pk}}\beta_0 = \frac{\beta_0}{M},\ \beta_1 = -\frac{\eta_1 * A_1 * RT}{\delta_1 * V_{pk}} - \frac{\eta_0 * A_0 * RT}{\delta_0 * V_{pk}} = -\frac{1+N}{MN}\beta_0,\ C = \frac{\eta_1 * A_1 * RT}{\delta_1 * V_{pk}} = \frac{\beta_0}{MN}$,

and $M = \frac{V_{pk}}{V_{t,h}}$ $, and\ N = \frac{A_0}{A_1} * \frac{\left(\frac{\eta_0}{\delta_0}\right)}{\left(\frac{\eta_1}{\delta_1}\right)}$

To solve Eqs. (S12-13), we introduce γ to satisfy for [γ*(S12) + (S13)] so that:

$$\frac{d\left(\gamma P_{in} + P_{pk}\right)}{dt} = (\gamma\alpha_0 + \alpha_1)P_{in} + (\gamma\beta_0 + \beta_1)P_{pk} + CP_{env}, \quad \text{(S14)}$$

$$and\ \frac{\gamma\alpha_0 + \alpha_1}{\gamma\beta_0 + \beta_1} = \gamma \quad \text{(S14')}$$

Solving Eq. (S14') gives us:

$$\beta_0\gamma^2 + (\beta_1 - \alpha_0)\gamma - \alpha_1 = 0 \quad \rightarrow \quad \gamma = \frac{(\alpha_0 - \beta_1) \pm \sqrt{(\alpha_0 - \beta_1)^2 + 4\alpha_1\beta_0}}{2\beta_0}, \quad i.e.\quad \gamma_+\ and\ \gamma_- \quad \text{(S15)}$$

Then Eq. (S15) can be changed to:

$$\frac{d\left(\gamma P_{in} + P_{pk}\right)}{dt} = \frac{d\left(\gamma P_{in} + P_{pk} + \frac{CP_{env}}{\gamma\beta_0 + \beta_1}\right)}{dt} = (\gamma\beta_0 + \beta_1)\left(\gamma P_{in} + P_{pk} + \frac{CP_{env}}{\gamma\beta_0 + \beta_1}\right) \quad \text{(S16)}$$

For both $\gamma_\pm$,

$$(\gamma_\pm\beta_0 + \beta_1) = \frac{(\alpha_0 + \beta_1) \pm \sqrt{(\alpha_0 - \beta_1)^2 + 4\alpha_1\beta_0}}{2} = \frac{-\beta_0\left(1 + \frac{1+N}{MN}\right) \pm \beta_0\sqrt{\left(-1 + \frac{1+N}{MN}\right)^2 + 4\frac{1}{M}}}{2}$$

$$= -\frac{\beta_0}{2MN}\left[(MN + N + 1) \mp \sqrt{(MN + N + 1)^2 - 4MN}\right] \quad \text{are negative.} \quad \text{(S17)}$$

Then the Eq. (S16) can be solved as:

$$\frac{\gamma_{\pm}P_{in}(t)+P_{pk}(t)+\frac{CP_{env}}{\gamma_{\pm}\beta_0+\beta_1}}{\gamma_{\pm}P_{in}(0)+P_{pk}(0)+\frac{CP_{env}}{\gamma_{\pm}\beta_0+\beta_1}} = e^{(\gamma_{\pm}\beta_0+\beta_1)t}$$, or

$$\gamma_{\pm}P_{in}(t) + P_{pk}(t) + \frac{CP_{env}}{\gamma_{\pm}\beta_0+\beta_1} = \left[\gamma_{\pm}P_{in}(0) + P_{pk}(0) + \frac{CP_{env}}{\gamma_{\pm}\beta_0+\beta_1}\right] * e^{(\gamma_{\pm}\beta_0+\beta_1)t} \quad \text{(S18)}$$

From Eq. (S18), when $t \to \infty, e^{(\gamma_{\pm}\beta_0+\beta_1)t} \to 0;\ P_{in}(t) and\ P_{pk}(t) \to P_{env},$ so we have:

$$\gamma_{\pm}P_{in}(\infty) + P_{pk}(\infty) + \frac{CP_{env}}{\gamma_{\pm}\beta_0+\beta_1} = 0,\ \ i.e.\ \ (\gamma_{\pm} + 1) = -\frac{C}{\gamma_{\pm}\beta_0+\beta_1} \quad \text{(S19)}$$

Then by eliminating either $P_{pk}(t)$ or $P_{in}(t)$ using Eq. (S18), we have:

$$(\gamma_+ - \gamma_-)P_{in}(t) - (\gamma_+ - \gamma_-)P_{env} = \Big[\big(\gamma_+P_{in}(0) + P_{pk}(0) - (\gamma_+ + 1)P_{env}\big)e^{-\frac{C}{\gamma_++1}t} -$$

$$\big(\gamma_-P_{in}(0) + P_{pk}(0) - (\gamma_- + 1)P_{env}\big)e^{-\frac{C}{\gamma_-+1}t}\Big] \quad \text{(S20a)}$$

$$(\gamma_+ - \gamma_-)P_{pk}(t) - (\gamma_+ - \gamma_-)P_{env} = \Big[-\gamma_-\big(\gamma_+P_{in}(0) + P_{pk}(0) - (\gamma_+ + 1)P_{env}\big)e^{-\frac{C}{\gamma_++1}t} +$$

$$\gamma_+\big(\gamma_-P_{in}(0) + P_{pk}(0) - (\gamma_- + 1)P_{env}\big)e^{-\frac{C}{\gamma_-+1}t}\Big] \quad \text{(S20b)}$$

After tube sealing, if we have $P_{in}(0) = P_{pk}(0)$, then the normalized relative vacuums for the tube and the intermediate container space can be deduced from Eqs. (S20a, b) as:

$$\Delta P_{in}'(t) = -\frac{P_{in}(t)-P_{env}}{P_{in}(0)-P_{env}} = -\frac{(\gamma_++1)e^{-\frac{C}{\gamma_++1}t}-(\gamma_-+1)e^{-\frac{C}{\gamma_-+1}t}}{(\gamma_+-\gamma_-)} \quad \text{(S21a)}$$

$$\Delta P_{pk}'(t) = -\frac{P_{pk}(t)-P_{env}}{P_{pk}(0)-P_{env}} = -\frac{-\gamma_-(\gamma_++1)e^{-\frac{C}{\gamma_++1}t}+\gamma_+(\gamma_-+1)e^{-\frac{C}{\gamma_-+1}t}}{(\gamma_+-\gamma_-)} \quad \text{(S21b)}$$

The β_0 in Eq. (S12) turns out to be the inverse of the tube gas leaking time constant, i.e. $\beta_{0,i} = \frac{1}{t_i}$, i=n, o. Then from Eq. (S21a), the normalized relative tube vacuum for nitrogen and oxygen becomes:

$$\Delta P_i{}'(t) = -\frac{P_{in,i}(t)-P_{env}}{P_{in,i}(0)-P_{env}} = -\frac{(\gamma_+ +1)e^{-\frac{t}{(\gamma_+ +1)MN*t_i}}-(\gamma_- +1)e^{-\frac{t}{(\gamma_- +1)MN*t_i}}}{(\gamma_+ -\gamma_-)} \quad \text{, i=n, o} \qquad \text{(S22)}$$

Similar to Eq. (S5), the overall normalized relative tube vacuum for air should be:

$$\Delta P'(t) = -0.785\Delta P_n{}'(t) - 0.215\Delta P_o{}'(t) \qquad \text{(S23)}$$

With Eqs. (22-23), we can plot $\Delta P'(t)$ over time if $\gamma_\pm$, M, N and t_i are known. $M = \frac{V_{pk}}{V_{t,h}}$, and V_{pk} and $V_{t,h}$ can be measured experimentally. $N = \frac{A_0}{A_1} * \frac{\left(\frac{\eta_0}{\delta_0}\right)}{\left(\frac{\eta_1}{\delta_1}\right)} = \frac{t_1}{t_0}$ requires not only geometry but also barrier data. $\gamma_\pm$ are not independent variables. Because $\alpha_0 = -\beta_0, \alpha_1 = \frac{\beta_0}{M},\ \beta_1 = -\frac{1+N}{MN}\beta_0,\ C = \frac{\beta_0}{MN},\ \gamma_\pm$ related values can be expressed by M and N as:

$$\gamma_\pm = \frac{(\alpha_0-\beta_1)\pm\sqrt{(\alpha_0-\beta_1)^2+4\alpha_1\beta_0}}{2\beta_0} = \frac{(N+1-MN)\pm\sqrt{(N+1-MN)^2+4MN^2}}{2MN}$$

$$\gamma_\pm + 1 = \frac{(N+1+MN)\pm\sqrt{(N+1-MN)^2+4MN^2}}{2MN} = \frac{(N+1+MN)\pm\sqrt{(N+1+MN)^2-4MN}}{2MN}$$

$$\gamma_+ - \gamma_- = \frac{\sqrt{(N+1-MN)^2+4MN^2}}{MN} = \frac{\sqrt{(N+1+MN)^2-4MN}}{MN} \qquad \text{(S24)}$$

To get an idea of how good the vacuum bag sealing approach could be for VacuStor tube application, we estimated M and N for the ARY 3-mil vacuum bag sealing of 96 empty glass VacuStor tubes. We used $V_{t,h}$ 1.042x96= 100 ml and V_{pk} 275.5 ml (from the tube rack and tube geometry) that gave a M value of 2.755. We also used A_0 0.5*96=48 cm^2, A_1 424.4 cm2 (from tube rack geometry), δ_0 1.1 mm, δ_1 0.0762 mm, η_0 and η_1 for oxygen 1129 and 3.66 cm^3-mm/m^2-day-atm that gave a N value of 2.417. η_1 for nitrogen is unknown. For simplicity, it was assumed a value that made the η_0/η_1 ratio the same as oxygen, i.e. the N value for nitrogen was also 2.417. Finally, we used the average SepraSeal leaking time constants of 54.46 and 18.47 days for nitrogen and oxygen respectively. The vacuum decay curve was plotted in the main text in Fig 5B.

S4. Self-collection educational video and preliminary testing of blood volume by a BD Microtainer Blue lancet

An educational video was recorded to show the self-collection process. The prototype collector used in the video had two 100 µl capillaries, which was different from the 50 µl used in the self-collection testing study. This difference was explained to the donors before their collections. The educational video is attached as a separate file as part of the supporting information.

Preliminary results of blood volume that can be generated from a single prick by a BD Microtainer Blue lancet are shown in Fig S2 below. The cause of two donors with less collected blood using the handwarmer is unknown, and could be related to piercing location, skin condition and lancet variations.

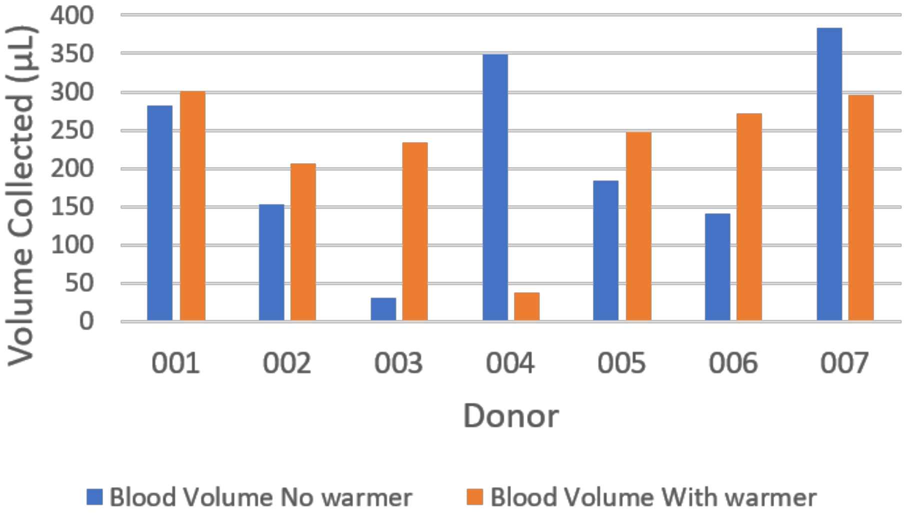

Fig S2 BD Microtainer Blue lancet blood volume with and without a hand warmer from 7 donors.

S5. Gene expression assay

RT-PCR comparison of gene expression across different donors

Gene expression in terms of fold change after irradiation compared to un-irradiated control blood of individual donor is shown in Table S1 below. No significant difference was found in terms of gene expression levels between traditionally and VacuStor collected samples.

Table S1 Comparison of gene expression across different donors for traditionally collected samples and self-collector collected samples.

	MDM2		***FDXR***		***BAX***		***CDKN1A***		***DDB2***	
	**Trad.**	**Self-C**	**Trad.**	**Self-C**	**Trad.**	**Self-C**	**Trad.**	**Self-C**	**Trad.**	**Self-C**
DONOR1	1.99	1.98	2.03	2.45	2.32	2.72	4.51	6.0	1.17	1.01
DONOR2	1.96	2.01	1.85	2.00	2.27	1.93	3.82	4.63	1.27	1.15
DONOR3	2.05	1.9	1.92	2.01	2.01	1.88	3.91	3.96	1.13	2.47
Mean	***2***	***1.96***	***1.94***	***2.15***	***2.20***	***2.17***	***4.08***	***4.86***	***1.19***	***1.54***
Std. Dev.	***0.045***	***0.056***	***0.096***	***0.25***	***0.16***	***0.47***	***0.37***	***1.03***	***0.07***	***0.80***
P-value (T-test)	**0.361**		**0.227**		**0.922**		**0.285**		**0.495**	

<u>*DxDirect assay*</u>

For DxDirect gene expression assay, we used the VacuStor system to collect blood samples irradiated with either 0 or 5 Gy of X-ray for gene-expression analysis using a non-enzymatic chemical ligation process (DxDirect, from Dxterity Diagnostics, CA), and compared the performance with benchtop blood handling by pipetting. Purchased blood was used for convenience. Fig S3 shows the results of 5 Gy gene expression level normalized by 0 Gy for 4 radiation dose-sensitive genes reported in the literature. Gene expression levels were detected for all 4 genes, and the radiation induced expression showed no significant difference between traditional benchtop pipetting process and the VacuStor collection, indicating that it is suitable for biodosimetry gene expression analysis.

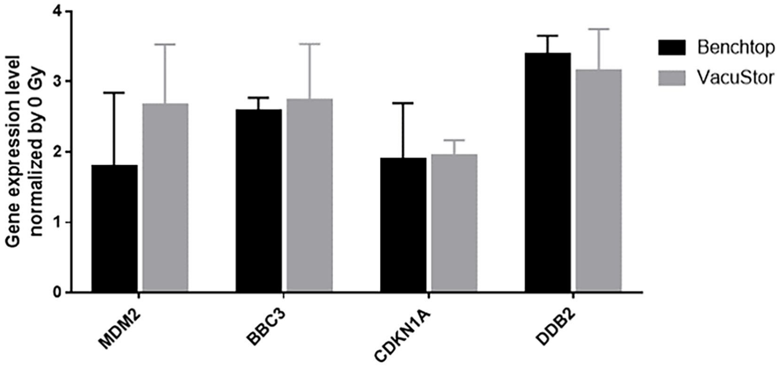

Fig S3 Gene expression comparison by DxDirect chemistry. Levels at 5 Gy normalized by those at 0 Gy for 4 radiation sensitive genes (MDM2, BBC3, CDKN1A, and DDB2) using blood samples handled by benchtop procedure and VacuStor system.

Human blood was purchased from BioChemed Services (Winchester, VA), mixed with RPMI/10% fetal bovine serum/1% penicillin-streptomycin (blood:media=1:1), and cultured in two 6-well plates in a CO_2 incubator at 37°C for 3-4 hours before irradiation. Irradiation was performed using a cabinet X-ray machine (X-RAD 320, Precision X-Ray Inc., North Branford, CT) at 320 kVp and 12.5 mA with a 2 mm Al filter. The source-to-axis distance was 42 cm. The beam was calibrated using a UNIDOS E PTW T10010 electrometer and TW30010-1 ion chamber, with measurement done in air, for a 15 cm x 15 cm field size. A total dose of 0 or 5.0 Gy was delivered to the 6-well plates at a dose-rate of 3 Gy/min. The samples were then cultured at 37°C for another 24 hours.

A commercially available assay chemistry test (DxDirect, DxTerity Diagnostics, Rancho Dominguez, CA) was used for multiplex gene expression analysis that combines a robust chemical ligation process and a sample stabilization buffer solution (http://dxterity.com/dx_direct.php). 100 µl of sample were collected from the 6-well plates using the capillary-needle assembly and transferred into VacuStor tubes containing 200 µl of DxCollect stabilization buffer. For benchtop controls, 100 µl of diluted blood were directly pipetted into micro-centrifuge tubes containing 200 µl of DxCollect stabilization buffer. Samples were immediately stored at -20°C until analysis. Three independent biological replicates were processed for each condition. The analysis followed manufacture suggested protocols. The final nucleic acid products were analyzed by capillary electrophoresis using ABI 3130xl genetic analyzer from ThermoFisher Scientific Inc. Each nucleic acid product was assayed in duplicate.

For data analysis, fluorescence values of peak height representing each gene were first log-transformed with a base of 2. Gene expression levels were then normalized by the non-radiation-responsive genes MRPS5, MRPS18, and CDR2, as specified in the assay kit, followed by the normalization of 5 Gy gene expression levels by the 0 Gy levels. Analyses were performed with GraphPad Prism version 7.00 for Windows (GraphPad Software Inc., La Jolla, CA).
